# Admission clinical characteristics and early clinical outcomes among acute ischemic stroke patients

**DOI:** 10.7555/JBR.26.20110129

**Published:** 2012-04-18

**Authors:** Xin Gao, Jintao Zhang, Ying Peng, Huanqing Fan, Mei Chen, Tan Xu, Yonghong Zhang

**Affiliations:** a Department of Epidemiology, School of Public Health, Medical College of Soochow University, Suzhou, Jiangsu 215123, China;; b Department of Neurology, The 88^th^ Hospital of PLA, Taian, Shandong 271000, China.

**Keywords:** acute ischemic stroke, clinical characteristics, death, neurological deficiency, discharge outcome

## Abstract

The purpose of the present study was to investigate the association between admission clinical characteristics and outcomes at discharge among acute ischemic stroke patients in the Chinese population. A total of 2,673 patients with acute ischemic stroke were included in the present study. The clinical characteristics at admission and other study variables were collected for all patients. The study outcome was defined as neurological deficiency (National Institute of Health Stroke Scale score ≥10) at discharge or in-hospital death. Compared with the subjects without neurological deficiency at discharge or in-hospital death, the subjects with neurological deficiency at discharge or in-hospital death had a significantly higher prevalence of hyperglycemia or history of atrial fibrillation at admission. Age ≥ 80 years, hyperglycemia, hypertension, and history of atrial fibrillation were significantly associated with neurological deficiency at discharge or in-hospital death after adjustment for other variables. It is concluded that old age (≥80 years), hyperglycemia, hypertension and history of atrial fibrillation are significantly associated with neurological deficiency at discharge or in-hospital death among patients with acute ischemic stroke.

## INTRODUCTION

Stroke has become one of the major causes of death and disability all over the world[1]. According to the data reported, the prevalence of stroke was 2.9% during 2003-2006 in the USA, and increased to 3.9% in 2008[2]. It was estimated that the total number of stroke patients was 7 million in China in 2004[1]. In the past few years in China, the number of stroke patients has risen following rapid economic growth, an increase in life expectancy, and changes in lifestyle[3],[4]. The burden of stroke is particularly high in China. A largesample prospective cohort study in China indicated that cerebrovascular disease accounted for 21.3% of all causes of death, and ranked as the second leading cause of death in men and the third leading cause of death in women[5]. The Sino-MONICA-Beijing Project suggested that there was a significant increase in incidence of ischemic stroke, which is an important feature of epidemiological transition of stroke in China[6]. A cross-sectional study by Zhang *et al*.[7] with 16,031 subjects who had strokes in China showed that the proportion of ischemic stroke was higher than that of any other stroke subtypes.

Clarifying the relationship between admission clinical characteristics and outcomes at discharge will be helpful for treatment and improvement of life quality and recovery among acute ischemic stroke patients. However, there is little knowledge on this relationship in the Chinese population till now. We examined the impact of admission clinical characteristics on the outcomes at discharge among patients with acute ischemic stroke.

## MATERIALS AND METHODS

### Subjects

A total of 2,868 acute ischemic stroke patients were recruited from four hospitals in three cities of Taian, Weifang and Zibo in Shandong Province, China. The stroke patients who were not hospitalized and died outside hospitals were not included in our study. All participants with a clinical diagnosis of acute ischemic stroke were admitted to the four hospitals from January 1, 2006 to December 31, 2008. Acute ischemic stroke was confirmed by computed tomography (CT) scan or magnetic resonance imaging (MRI). A team of investigators including neurologists reviewed all data about the selected patients. One hundred ninety-five patients were excluded from the present study because of missing related data. A total of 2,673 remaining subjects were included in this analysis. This study was approved by the Ethics Committee of School of Radiation Medicine and Public Health of Soochow University.

### Data collection

The data on demographic characteristics, lifestyle risk factors, medical history, family history of diseases, blood pressure, clinical laboratory tests and complications during acute phase, and the data on in-hospital death and National Institutes of Health Stroke Scale (NIHSS) score at discharge were obtained from medical records. Cigarette smokers were defined as having smoked at least one cigarette per d for 12 months or more. The amount and type of alcohol consumed during the past year was collected. Alcohol consumption was defined as consuming one or more alcoholic drinks per d during the last year. Blood pressure (BP) was measured in the first 30 min after admission while the study participants were in the supine position using a standard mercury sphygmomanometer according to a standard protocol[8]. The first and fifth Korotkoff sounds were recorded as the systolic blood pressure (SBP) and diastolic blood pressure (DBP), respectively. The definition of hypertension at admission was SBP of ≥ 140 mmHg and/or DBP of ≥ 90 mmHg[9].

Plasma glucose was measured using a modified hexokinase enzymatic method. Hyperglycemia was defined as fasting plasma glucose (FPG) of ≥ 6.1 mmol/L[10]. Total cholesterol (TC), high-density lipo-protein cholesterol (HDL-C), and triglycerides (TG) were analyzed enzymatically on a Beckman Synchron CX5 Delta Clinical System (Beckman Coulter, Inc., Fullerton, CA, USA) using commercial reagents[11]. Low-density lipoprotein cholesterol (LDL-C) levels were calculated by using Friedewald equation[12]. Subjects with any one of the following indexes were considered to have dyslipidemia: high TC (TC ≥5.18 mmol/L), high TG (TG ≥1.7 mmol/L), high LDL-C (LDL-C ≥3.37 mmol/L), low HDL-C (HDL-C < 1.04 mmol/L)[13]. Plasma glucose and lipidogram were measured in the first 24 h after admission on an empty stomach. If a patient died in the hospital, a study staff recorded the death on the event form and obtained the death certificate. If a patient survived the acute ischemic stroke, the study neurologists conducted a comprehensive clinical evaluation using NIHSS at discharge. The study outcome was defined as neurological deficiency (NIHSS ≥10)[14] at discharge or inhospital death.

### Statistical analysis

Patients were divided into two groups, one with study outcome and the other without study outcome[15]-[17]. The demographic characteristics and clinical characteristics of the subjects in the two groups were described in the following ways. Agespecific rates were analyzed by age groups (≤49, 50 to 64, 65 to 79, ≥80 years). The mean and standard deviation were used to describe continuous variables while proportion was used to describe categorical variables on admission. Student *t*-test was used to compare the differences of means among groups, while χ^2^-test was used for testing the differences of proportions. Logistic regression models were used to evaluate the odds ratio (OR) of the study outcomes related to sex, age, cigarette smoking, alcohol drinking, dyslipidemia, hyperglycemia, hypertension, history of hypertension, history of coronary heart disease, history of atrial fibrillation, history of rheumatic heart disease, and history of cardio-cerebrovascular diseases and then all of the variables were adjusted among each other. All *P*-values (2-sided test) < 0.05 were considered statistically significant. The database was established using the EpiData package, and the statistical software SPSS version 16.0 (SPSS Inc., Chicago, IL, USA) was used for all statistical analyses.

## RESULTS

Unadjusted ORs and 95% confidence intervals (95% CIs) of the study outcomes with baseline characteristics on admission among the subjects are shown in ***Table 1***. There were 2,468 subjects (92.3%) without study outcomes and 205 subjects (7.7%) with study outcomes (186 patients with NIHSS≥10 at discharge and 19 in-hospital deaths) in 2,673 ischemic stroke participants. The study outcome group tended to have a higher prevalence of hyperglycemia, history of atrial fibrillation, and history of rheumatic heart disease compared with the group without study outcome (all *P* values < 0.05). The study outcome group also tended to have a higher rate of high LDL-C in comparison to those without study outcomes, but no significant difference was observed between the two groups (*P* = 0.055). There were no statistically significant differences in gender, cigarette smoking, alcohol drinking, high TC, high TG, low HDL-C, hypertension, history of hypertension, history of coronary heart disease, and family history of cardio-cerebrovascular disease between the two groups (all *P* values > 0.05). Singlefactor logistic regression models showed that the ORs of the study outcomes in the subjects aged 50-64 and ≥80 years were 1.935 and 2.467, respectively (both *P* values < 0.05), compared with those aged < 49 years. The study outcome was significantly associated with hyperglycemia, history of atrial fibrillation and history of rheumatic heart disease, with ORs of 2.797, 3.807 and 3.376, respectively (all *P* values < 0.05).

***Table 2*** presents the multivariate adjusted ORs and 95% CIs of the study outcomes related to admission clinical characteristics. After adjustment for all other variables, the OR of the study outcome for age ≥ 80 years was 2.941 compared to those age < 49 years; the association of the study outcome with hyperglycemia and history of atrial fibrillation was still significant (all *P* values < 0.05); however, it was not significant for history of rheumatic heart disease (*P* > 0.05). The OR of the study outcome for hypertension was significant after adjustment for all other variables (*P* < 0.05). Either in unadjusted or in multivariate adjusted models, the ORs (1.462 and1.655, respectively) of the study outcome with high LDL-C were, although not statistically significant, in the expected direction.

**Table 1:** Unadjusted odds ratio and 95% confidence intervals of study outcome with baseline characteristics upon admission in patients with acute ischemic stroke

Variables	Without study outcome	With study outcome	OR	95% CI	*P*-value
N	2,468	205			–
					0.729
Men, n(%)	1,559(63.2)	127(62.0)	1.053	(0.785,1.413)	0.088
Age (year, Mean±SD)	64.180±11.882	65.650±11.526			0.026
0-49, n(%)	297(12.0)	14(6.8)	1.000	–	0.075
					0.009
50-64, n(%)	888(36.0)	81(39.5)	1.935	(1.081, 3.464)	0.780
65-79, n(%)	1,065(43.2)	85(41.5)	1.693	(0.948, 3.023)	0.249
					0.383
> 80, n(%)	215(8.7)	25(12.2)	2.467	(1.253, 4.856)	0.184
					0.145
Cigarette smoking, n(%)	615(24.9)	50(24.4)	0.954	(0.684, 1.330)	0.055
Alcohol drinking, n(%)	598(24.2)	43(21.0)	0.815	(0.575, 1.155)	< 0.001
					0.154
High TC, n(%)	875(35.5)	78(38.0)	1.145	(0.845, 1.551)	0.294
High TG, n(%)	652(26.4)	45(22.0)	0.789	(0.556, 1.120)	0.401
					< 0.001
Low HDL-C, n(%)	436(17.7)	26(12.7)	0.718	(0.458, 1.124)	0.033
High LDL-C, n(%)	432(17.5)	43(21.0)	1.462	(0.989, 2.159)	0.153
Hyperglycemia, n(%)	792(32.1)	116(56.6)	2.797	(2.078, 3.767)	
Hypertension, n(%)	1,700(68.9)	151(73.7)	1.263	(0.915, 1.744)	
History of hypertension, n(%)	1,515(61.4)	119(58.0)	0.857	(0.642, 1.144)	
History of coronary heart disease, n(%)	401(16.2)	38(18.5)	1.171	(0.810, 1.692)	
History of atrial fbrillation, n(%)	64(2.6)	19(9.3)	3.807	(2.233, 6.489)	
History of rheumatic heart disease, n(%)	18(0.7)	5(2.4)	3.376	(1.241, 9.189)	
Family history of cardio-cerebrovascular disease, n(%)	286(11.6)	17(8.3)	0.690	(0.414, 1.151)	

SD: standard deviation; TC: total cholesterol; HDL-C: high density lipoprotein cholesterol; TG: triglycerides; LDL-C: low density lipoprotein cholesterol.

**Table 2:** Multivariate adjusted odds ratio and 95% confidence intervals of study outcome with baseline characteristics upon admission in patients with acute ischemic stroke

Variables	Adjusted* OR	95% CI	*P*-value
Men	1.161	(0.730, 1.866)	0.518
Age (year)			
0-49	1.000	–	–
50-64	1.146	(0.546, 2.405)	0.718
65-79	1.268	(0.608, 2.646)	0.526
≥80	2.914	(1.225, 6.932)	0.016
Cigarette smoking	1.420	(0.761, 2.650)	0.270
Alcohol drinking	0.947	(0.499, 1.800)	0.869
High TC	0.700	(0.392, 1.250)	0.228
High TG	0.820	(0.508, 1.324)	0.417
Low HDL-C	0.740	(0.451, 1.215)	0.234
High LDL-C	1.655	(0.941, 2.910)	0.080
Hyperglycemia	2.820	(1.877, 4.236)	< 0.001
Hypertension	1.724	(1.043, 2.849)	0.034
History of hypertension	0.953	(0.621, 1.461)	0.825
History of coronary heart disease	0.767	(0.429, 1.371)	0.371
History of auricular fbrillation	3.156	(1.477, 6.746)	0.003
History of rheumatic heart disease	0.499	(0.056, 4.456)	0.533
Family history of cardio-cerebrovascular disease	0.913	(0.489, 1.706)	0.775

*adjusted each other among all the variables. TC: total cholesterol; HDL-C: high density lipoprotein cholesterol; TG: triglycerides LDL-C: low density lipoprotein cholesterol.

## DISCUSSION

Our study indicated that old age (age ≥80 years), hyperglycemia, hypertension, and history of atrial fibrillation were significantly associated with neurological deficiency at discharge or in-hospital death among acute ischemic stroke patients, and the association of the study outcome with high LDL-C was in the expected direction but not significant.

Fonarow *et al*. [18] reported that older patients with ischemic stroke had higher in-hospital mortality than younger patients. Other studies found that advanced age (≥ 80 years) was associated with poor short-term outcome[19]-[21]. Consistent with previous reports[18]-[21], in both univariate logistic regression and multivariate logistic regression analysis, very old subjects (age ≥80 years) in our study had a significant higher incidence of the study outcome than the younger patients (≤49 years).

Our findings indicated that hyperglycemia was associated with study outcome in both unadjusted and multivariate adjusted analysis. Compared with those without study outcomes, subjects with study outcomes had a significantly higher prevalence of hyperglycemia. Logistic regression models showed that after adjustment for other variables, the risk of study outcome in subjects with hyperglycemia at admission was 2.82 times more than those without hyperglycemia. A retrospective study based on 445 stroke patients without a history of diabetes mellitus indicated that blood glucose increase after the onset of acute stroke was related to the severity of the stroke[22]. Some previous studies also showed that hyperglycemia was related to a poor outcome of acute ischemic stroke[23]-[25]. Williams *et al*.[23] thought that admission hyperglycemia was associated with short- and long-term mortality among patients with acute ischemic stroke. The relationship between hyperglycemia and severity of acute ischemic stroke can be explained by increased lactate production in the ischemic region, and the production generates hydrogen ions and deranges the intracellular pH homeostasis, which may seriously affect some reactions and enzyme systems, the integrity of which is necessary for cellular viability[26]-[29].

Some, but not all, previous studies suggested that hypertension at admission was associated with poor outcome among acute ischemic stroke patients[30]-[34]. Vemmos *et al*.[30] reported that there was a U-shaped relationship between admission blood pressure and mortality in patients with acute ischemic stroke, indicating that acute ischemic stroke patients with extremely high and low admission blood pressure values had a higher early and late mortality. Leonardi-Bee *et al*.[31] also indicated that there was a U-shaped relationship between baseline blood pressure and poor outcome of ischemic stroke. It was also suggested that higher blood pressures were associated with a significantly increased risk of recurrent ischemic stroke within 14 d among ischemic stroke patients. However, some other studies did not reveal a significant association between admission blood pressure and case-fatality rate among acute ischemic stroke patients[32]-[34]. Our present study supported the notion that hypertension at admission was related to neurological deficiency at discharge or in-hospital death among acute ischemic stroke subjects.

In addition, our study showed a strong relationship between history of atrial fibrillation and study outcome. Compared with those without a history of atrial fibrillation, subjects with a history of atrial fibrillation had more than three-fold increase in the risk of study outcome after adjustment for other variables. There were some studies reporting the relationship between atrial fibrillation and poor outcome of stroke[35]-[39]; all these reports consistently suggested that a history of atrial fibrillation was a risk factor for a poor outcome of stroke. It has been reported that acute stroke patients with atrial fibrillation are generally more serious and have poorer outcome than patients without atrial fibrillation[38],[39]. In addition, our findings also showed that the OR (1.462) of study outcome with high LDL-C was in the expected direction, although not statistically significant. Zeljkovic *et al*.[40] reported that increased small dense LDL particles were associated with short-term mortality among acute ischemic stroke patients.

In summary, our findings suggest that treatment aimed at reducing plasma glucose and blood pressure in patients with hyperglycemia and hypertension, reducing the severity of atrial fibrillation, or lowering the level of LDL-C among acute ischemic stroke patients should be emphasized to reduce the risk of neurological deficiency or in-hospital death among acute ischemic stroke patients.
